# Editorial: Immunological mechanisms, clinical presentations, and outcomes of neurological toxicities induced by immune checkpoint inhibitors

**DOI:** 10.3389/fimmu.2026.1940136

**Published:** 2026-07-22

**Authors:** Alberto Vogrig, Sergio Muñiz-Castrillo

**Affiliations:** 1 Department of Medicine (DMED), University of Udine, Udine, Italy; 2 Clinical Neurology, Department of Head-Neck and Neurosciences, Azienda Sanitaria Universitaria Friuli Centrale (ASUFC), Udine, Italy; 3 Neurology Department, Hospital Universitario 12 de Octubre, Instituto de Investigación Sanitaria Hospital 12 de Octubre (Imas12), Madrid, Spain

**Keywords:** cancer immunotherapy, immune checkpoint inhibitors, neurological adverse events, neurological toxicities, paraneoplastic neurological syndromes

Immune checkpoint inhibitors (ICIs) have fundamentally reshaped cancer therapy by harnessing adaptive immunity, particularly T cell responses, to achieve durable antitumor effects and meaningful improvements in survival (1). However, this therapeutic benefit comes at the cost of immune related adverse events (irAEs), which can affect virtually every organ. Among these, neurological toxicities (n-irAEs) represent a particularly challenging complication: although uncommon overall, occurring in approximately 1 to 3% of treated patients, they can cause severe and potentially irreversible harm when recognition or management is delayed (2, 3). Their clinical spectrum is broad, encompassing peripheral and central nervous system manifestations, the former being approximately three times more frequent, and, importantly, their pathophysiology remains incompletely understood (2, 4).

In this context, the role of neuronal antibodies further illustrates this complexity. Some patients harbor such antibodies without developing overt toxicity, as with Hu antibodies in small-cell lung cancer (SCLC); others, conversely, develop them yet without the pathogenicity characteristic of the classic disease, as with anti-Acetylcholine Receptor antibodies in ICI-associated myocarditis-myositis syndrome; and, finally, others lack detectable antibodies altogether, as is frequently observed in ICI-associated Guillain-Barré syndrome and meningoencephalitis (2, 5, 6).

This apparent paradox is analyzed in detail within the present Research Topic in Frontiers in Immunology. In a prospective cohort study of patients with SCLC, Rossi et al. found neuronal antibodies in approximately one third of patients at baseline, yet antibody positivity did not confer an increased risk of n-irAEs following ICI treatment. This dissociation is consistent with earlier observations that neuronal autoantibodies — Hu in particular — may be present in cancer patients reflecting a subclinical antitumor immune response rather than an active neuroimmune disorder (7). Compounding this complexity, anti-Hu–associated autoimmunity itself is phenotypically diverse, often multifocal, and may antedate cancer diagnosis by months to years (8).

Taken together, these observations argue against the binary use of autoantibody positivity as a predictive biomarker and call for a more contextually informed interpretation. The identity of the antibody and its immunological milieu matter considerably: anti-Hu positivity, for instance, has been associated with a higher burden of systemic irAEs even when overt neurological toxicity is absent (Rossi et al.). In the domain of paraneoplastic neurological syndromes (PNS), antibodies directed against intracellular antigens — so-called “high-risk” antibodies — correlate with more severe central nervous system involvement and poorer outcomes, consistent with the understanding that such antibodies serve as surrogates for a pathogenic, cytotoxic T cell–mediated immune response (Ren et al.), corroborating prior evidence in the field (3). The clinical implication is that the same serological finding may carry very different prognostic weight depending on the antigen target, tumor context, and the underlying immune effector mechanisms at play.

Molecular profiling is beginning to illuminate the tumor–immune interface that underlies these divergent autoimmune phenotypes. Genomic and transcriptomic analyses of SCLC have identified distinct immune gene expression signatures associated with each antibody-defined syndrome — anti-GABA_B_R versus anti-Hu, for example — suggesting that tumor-intrinsic genetic and epigenetic features actively shape the quality and specificity of the paraneoplastic immune response (9). This bidirectional relationship between tumor biology and immune phenotype has important implications, since the neurological toxicity profile of a given patient may, at least in part, be inscribed in the genomic architecture of their malignancy.

The neurological footprint of ICI therapy extends beyond classical paraneoplastic mechanisms to encompass subtler, often underrecognized manifestations. Notably, cerebral small vessel disease (CSVD) has emerged as a candidate immune-related adverse event, with associations to patient age, hyperlipidemia, and ICI-associated hypothyroidism (Wu et al.). These observations raise the possibility that sustained immune activation — even in the absence of frank autoimmunity — may promote chronic endothelial inflammation and progressive microvascular injury, adding a neurovascular dimension to the spectrum of ICI-related harm. As ICI-enabled survival extends into the long term, the cumulative vascular consequences of chronic immune activation — including an elevated risk of cerebrovascular events — deserve dedicated prospective study.

From a clinical standpoint, the phenotypic heterogeneity of n-irAEs continues to confound timely diagnosis and management. Real-world cohorts and systematic reviews converge on the finding that neuromuscular disorders, including myositis and inflammatory neuropathies, represent the most prevalent presentations (2, 10). However, the subset of ICI-triggered paraneoplastic-like syndromes disproportionately involves the central nervous system and is associated with substantially worse neurological outcomes, underscoring the importance of early serological and imaging evaluation in patients presenting with subacute encephalopathy, cerebellar dysfunction, or brainstem signs during ICI therapy (3).

Mechanistically, the pathogenesis of n-irAEs is probably multifactorial. Antigen mimicry between tumor neoantigens and neuronal epitopes, cytokine-driven neuroinflammation, and the breakdown of central immune tolerance all likely contribute to the diverse neurological syndromes observed clinically (Vogrig et al.). In this context, soluble biomarkers are gaining importance as potential early warning signals: serum neurofilament light chain (NfL), a marker of neuroaxonal injury, and specific cytokine profiles (e.g. increase of CXCL10 and CXCL13) are under active investigation as tools for prospective monitoring and risk stratification in patients receiving ICI therapy (Vogrig et al.).

The articles gathered in this Research Topic collectively articulate a compelling case for a precision immunotoxicology framework—one that moves beyond timely diagnosis toward prospective, biology-informed risk stratification. Achieving this will require longitudinal studies that integrate clinical phenotyping with multi-omic profiling of tumor, blood, and cerebrospinal fluid, alongside prospective biobanking and standardized outcome reporting across institutions. Ultimately, the goal is to identify, before the first infusion, which patients harbor the biological preconditions for severe neurological harm—enabling to individualize monitoring protocols, calibrate immunosuppressive responses, and ensure that the remarkable therapeutic gains of ICI therapy are not offset by preventable neurological injury.
