# Correction: Editorial: Immunological mechanisms, clinical presentations, and outcomes of neurological toxicities induced by immune checkpoint inhibitors

**DOI:** 10.3389/fimmu.2026.1960530

**Published:** 2026-09-01

**Authors:** Frontiers Production Office

**Affiliations:** Frontiers Media SA, Lausanne, Switzerland

**Keywords:** cancer immunotherapy, immune checkpoint inhibitors, neurological adverse events, neurological toxicities, paraneoplastic neurological syndromes

The title of this article was erroneously given as: “Editorial: Toward predictive and mechanistic approaches for precision neuroimmunotoxicology.” The correct title of the article is: “Editorial: Immunological mechanisms, clinical presentations, and outcomes of neurological toxicities induced by immune checkpoint inhibitors.”

The original version of this article has been updated.

